# Effects of melatonin-pretreated adipose-derived mesenchymal stem cells (MSC) in an animal model of spinal cord injury

**DOI:** 10.1186/s12868-022-00752-6

**Published:** 2022-11-16

**Authors:** Arvin Naeimi, Arash Zaminy, Naser Amini, Raziye Balabandi, Zoleikha Golipoor

**Affiliations:** 1 Student Research Committee, School of Medicine, Guilan University of Medical Sciences, Rasht, Iran; 2 Burn and Regenerative Medicine Research Center, Velayat Hospital, School of Medicine, Guilan University of Medical Sciences, Rasht, Iran; 3 Cellular and Molecular Research Center, Iran University of Medical Sciences, Tehran, Iran; 4 Cellular and Molecular Research Center, Faculty of Medicine, Guilan University of Medical Sciences, Rasht, Iran

**Keywords:** Melatonin, Adipose-derived mesenchymal stem cells, Mesenchymal stem cells, Spinal cord injury, Pretreatment, Animal model, SCI, ADSC, MSC

## Abstract

**Background:**

One of the most serious nervous system diseases is spinal cord injury(SCI), which is increasing for various reasons. Although no definitive treatment has yet been identified for SCI, one possible treatment is adipose-derived stem cells(ADSCs). However, a key issue in transplantation is improving cells’ survival and function in the target tissue. Melatonin(MT) hormone with antioxidant properties can prolong cell survival and improve cell function. This study investigates the pre-conditioning of ADSCs with melatonin for enhancing the engraftment and neurological function of rats undergoing SCI.

**Methods:**

42 male Sprague–Dawley rats were divided into six groups, including Control, Sham, Model, Vehicle, and Lesion treatments A and B. After acquiring white adipose tissue, stem cells were evaluated by flow cytometry. SCI was then applied in Model, Vehicle, A, and B groups. Group A and B received ADSCs and ADSCs + melatonin, respectively, 1 week after SCI, but the vehicle received only an intravenous injection for simulation; The other groups were recruited for the behavioral test. Immunohistochemistry(IHC) was used to assess the engraftment and differentiation of ADSCs in the SCI site. Basso, Beattie, and Bresnahan's score was used to evaluate motor function between the six groups.

**Results:**

Histological studies and cell count confirmed ADSCs implantation at the injury site, which was higher in the MT-ADSCs (P < 0.001). IHC revealed the differentiation of ADSCs and MT-ADSCs into neurons, astrocytes, and oligodendrocyte lineage cells, which were higher in MT-ADSCs. Functional improvement was observed in SCI + ADSCs and SCI + MT-ADSCs groups.

**Conclusion:**

The pre-conditioning of ADSCs with melatonin positively affects engraftment and neuronal differentiation in SCI but does not impact performance improvement compared to the ADSCs.

## Background

Spinal cord injury (SCI) is defined as damage to the spinal cord associated with temporary or permanent changes in the spinal cord, leading to sensory, motor, and autonomic deficits [1]. This condition is presently the most challenging traumatic neurological disorder to treat, accompanied by a substantial negative impact on the patient's mental, physical, and social well-being, as well as an enormous economic burden on both patient and society [1–3]. The incidence of SCI ranges from 20.7 to 83.0 per million annually in North America, and its incidence is growing worldwide, estimated at 10.4 to 83 cases per million [4]. Despite some advances in treatment strategies, there is still no gold standard treatment option for SCI [5]. Current treatments include spinal decompression surgery, pharmacological therapy for alleviating spasticity, and rehabilitation therapy. However, the resultant recovery from these options is very limited, prompting researchers to look for more advanced therapeutic options [6]. In this regard, stem-cell-based therapy, as a novel therapeutic option, has yielded promising results in improving nerve function and replacing damaged nerves [7]. To clarify the mechanisms by which stem cells improve nerve injury, it is better to look at SCI neurological outcomes. SCI is divided into two phases. The primary stage is defined by the mechanical destruction of nerve cells following physical force, leading to laceration, stretching, contusion, or compression of nerve cells [5, 8]. The damaged cells then enter the second phase, which is mainly characterized by the release of cytokines and inflammatory factors. In fact, the mechanical injury disrupts the blood flow, resulting in ischemia, electrolyte shifts, lipid peroxidation, and the generation of toxic metabolites and free radicals. The resultant cascade stimulates the production of inflammatory cytokines, chemokines, and fibroblasts' emergence, leading to scar formation [1, 9, 10]. Studies have shown that stem cells drive neuroprotection and nerve regeneration through the following mechanisms: Stem cells exert anti-inflammatory effects by downregulating the pro-inflammatory and pro-apoptotic genes, producing anti-inflammatory factors, and upregulating the expression of neuroprotective genes. In addition, they can be differentiated into neural and glial cells, replacing the damaged cells and forming interneurons, resulting in the formation of new synapses [2, 11]. Despite promising results achieved from stem cell therapy in SCI, finding an ideal stem cell type to treat nerve injuries poses a great challenge. This is mainly due to variations in the physiological and biological functions of different types of stem cells, the risk of post-transplant rejection, high costs, and the feasibility of access [12]. Accordingly, several stem cell types have been studied to find a treatment for SCI, including mesenchymal stem cell (MSC), embryonic stem cell (ESC), fetal-derived neural stem cells, and central nervous system stem cells. To date, MSC is the most commonly studied cell type in SCI [1]. MSCs are a group of heterogeneous cells derived from adipose tissue, bone marrow, and umbilical cord [13]. They possess three main features making them a suitable candidate for stem cell therapy. Firstly they can be differentiated into several types of cells, including adipocytes, osteoblasts, hepatocytes, myoblasts, and neuron-like cells both in vivo and in vitro; secondly, they can be located at the damaged sites by pursuing the chemotactic signals; thirdly, they are capable of producing numerous factors including cytokines, chemokines and growth factors, leading to neuroprotective and immunomodulatory behavior [14]. Finally, these cells, compared to other cell types, have low immunogenicity [15].

Bone marrow stem cell (BMSC) is a type of MSC extensively studied to treat SCI [1]. BMSCs are adult stem cells distributed widely in bone marrow with self-renewal and neuronal cell differentiation features. These cells release axonal regeneration proteins such as growth-associated protein 43 (GAP-43) as well as neurotrophic factors such as brain-derived neurotrophic factors (BDNF) and nerve growth factors (NGF) [16]. Although BMSC has been shown to be effective in SCI clinical trials, there are some limitations regarding administering these cells [17]. The procedure of BMSC extraction is invasive, painful, and low efficacious compared to adipose tissue-derived stem cells (ADSC), another source of MSC [18]. In addition, the application of BMSC is associated with ectopic migration and potential tumorigenicity [11]. On the other hand, studies have shown that ADSC has better performance than BMSC in nerve injury repair [19]. ADSC, similar to BMSC, are multipotent cells. Besides, they are more accessible than BMSC, obtainable in larger amounts from subcutaneous fat using conventional liposuction and have a higher proliferation rate [16, 18, 19].

Despite the promising clinical outcomes of stem cell therapy, there are some barriers to this therapeutic approach. A major obstacle is the rejection of cell engraftment in the target tissue [14]. It has been shown that 80 percent of cells die within 72 h after infusion [5]. This is partly due to hypoxia and oxidative stress in the injured tissue, which has been demonstrated to inhibit cell proliferation, adhesion, and migration at the graft site. In this regard, research shows that antioxidant pre-conditioning enhances engraftment efficacy and stimulates cell proliferation [2, 19].

One of the pre-conditioning antioxidants proven to promote cell viability is melatonin [20]. Melatonin is an endogenous hormone produced and secreted by the pineal gland. At the molecular level, melatonin acts as an antioxidant by removing reactive oxygen species (ROS) and reducing endoplasmic reticulum stress (ERS). It is also associated with angiogenesis and inflammatory pathways [21]. In addition, the melatonin pre-conditioning of ADSC and BMSC has been shown to increase cell viability and inhibit apoptosis under different conditions, including ischemic kidney, liver fibrosis, and neural stem cells [5, 20]. However, the results of studies on the role of melatonin pre-conditioning in ADSC for SCI treatment are still unclear. This study investigated the possible effect of melatonin on enhancing ADSC engraftment efficacy in SCI rat models.

## Results

### ADSCs isolation and culture

**Fig. 1:**
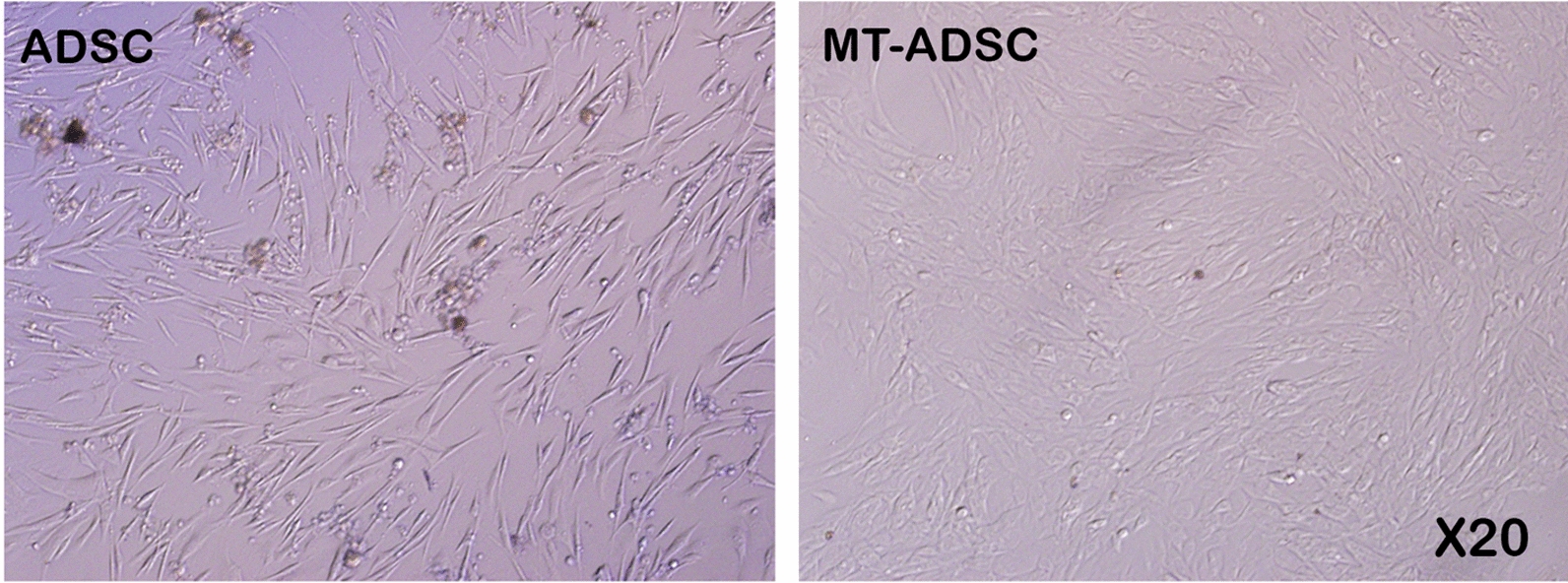
The morphology and density of ADSCs and MT-ADSCs. Undifferentiated ADSCs and undifferentiated MT-ADSCs display a flattened fibroblast-like morphology under phase-contrast microscopy. Within 4–5 days in culture medium, MT-ADSCs (80–90%) reached higher densities than ADSCs (40–50%); (Magnification: 20×)

Based on the isolation and culture results, MT-ADSCs (80–90%) reached higher densities than ADSCs (40–50%) within 4–5 days in the culture medium (Fig. 1).

### ADSCs flow cytometry analysis

**Fig. 2:**
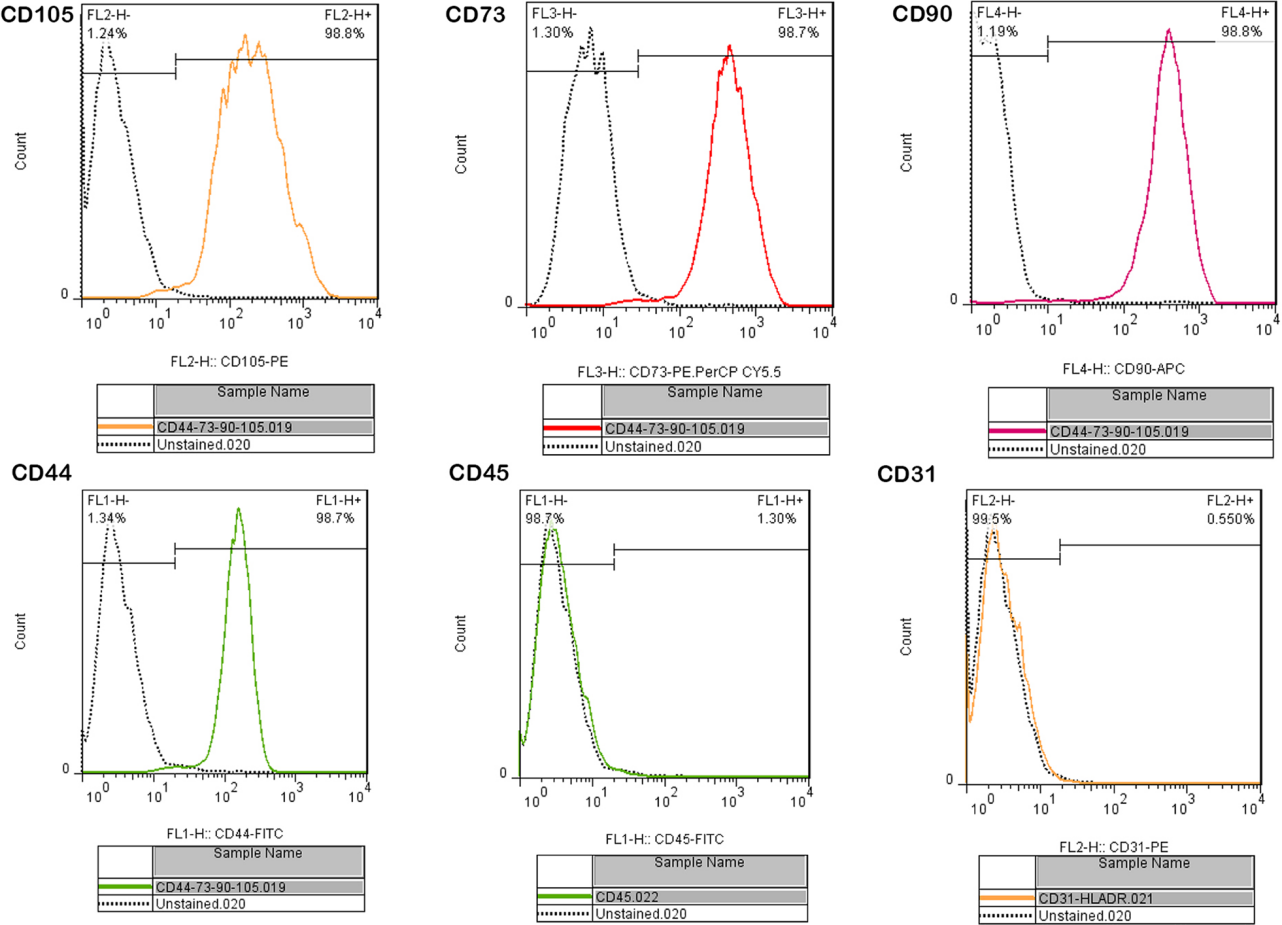
Isolation and identification of mouse adipose tissue-derived stem cells (ADSCs). The expression of ADSC surface markers was detected by flow cytometry so that they were positive for **A** CD105, **B** CD73, **C** CD90, and **D** CD44 and negative for **E** CD45 and **F** CD31

Flow cytometric analysis showed positive (CD44, CD90, CD105, and CD73) and negative (CD45 and CD31) cell surface markers indicating the mesenchymal nature of ADSCs (Fig. 2).

### Confirmation model

**Fig. 3:**
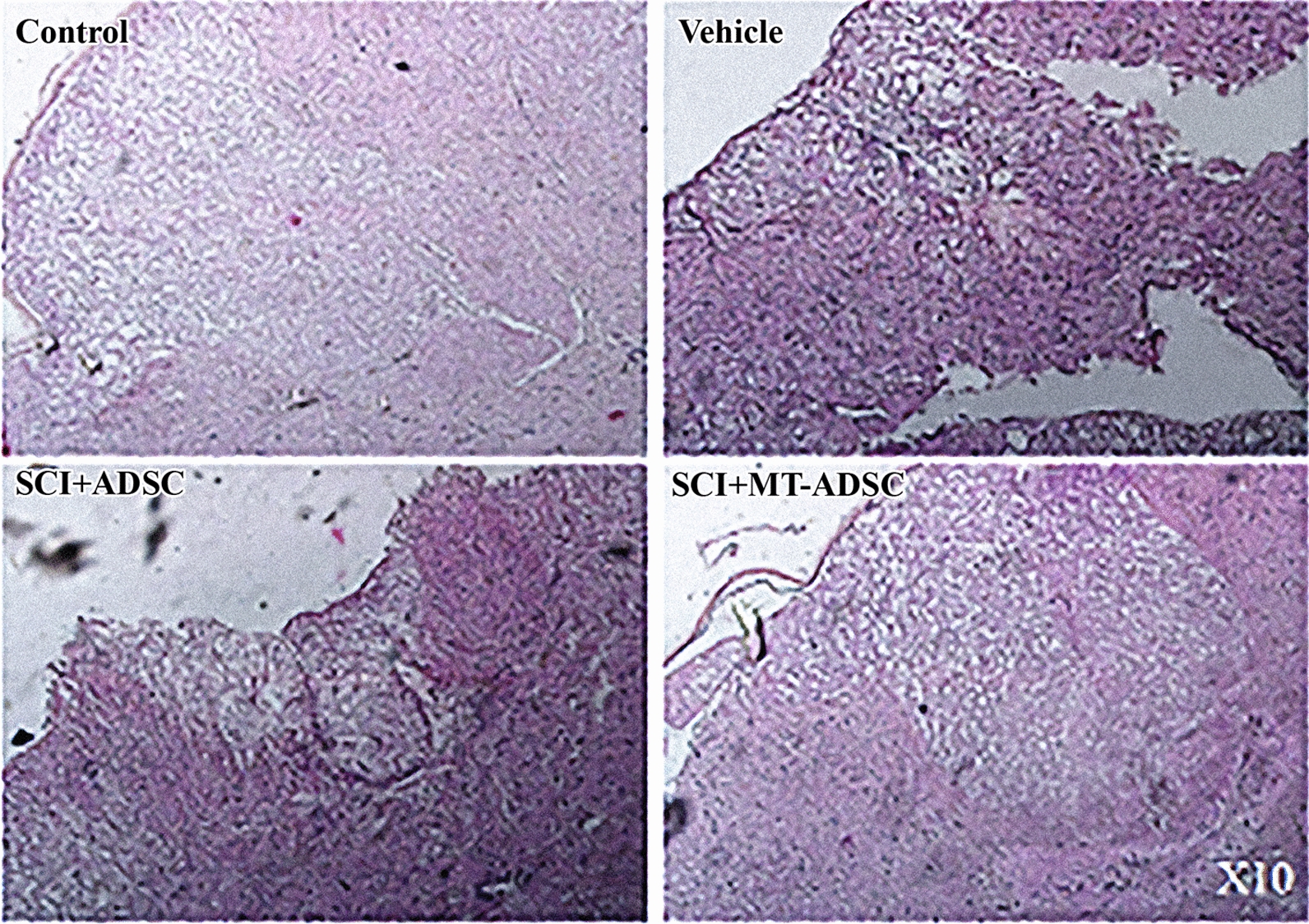
Injury confirmation model. H&E staining of spinal cord lesions revealed that the spinal injury site of the Control group was normal. However, the spinal injury site of the Vehicle (SCI + DMEM), SCI + ADSC, and SCI + MT-ADSC groups had hollow cystic cavities that confirmed the injury; (Magnification: 10×)

According to H&E staining, the presence of hollow cystic cavities at the SCI site of the SCI + DMEM, SCI + ADSCs, and SCI + MT-ADSCs groups confirmed the SCI model (Fig. 3).

### Presence of ADSCs and MT-ADSCs at the site of SCI

**Fig. 4:**
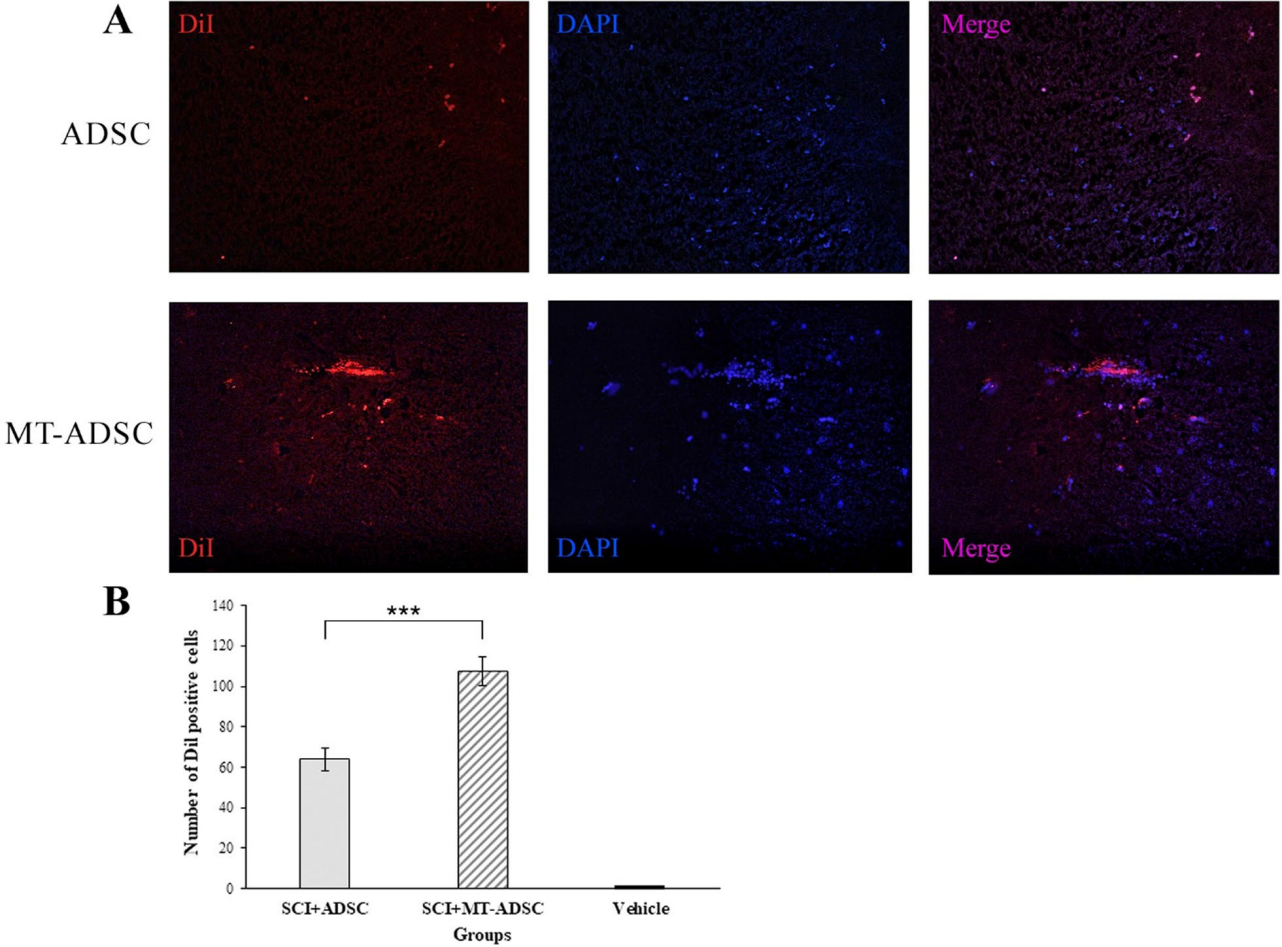
Evaluation of ADSCs and MT-ADSCs at the site of SCI. **A** At the site of injury: ADSCs and MT-ADSCs labeled with DiI; The nuclei of ADSCs and MT-ADSCs labeled with DAPI. **B** Comparison of the number of DiI positive cells between Vehicle (SCI + DMEM), SCI + ADSCs and SCI + MT-ADSCs groups demonstrated that the number of MT-ADSCs was higher than ADSCs at the SCI site (***P < 0.001); (Magnification: 20×)

Based on IHC analysis in the Vehicle (SCI + DMEM), SCI + MT-ADSCs, and SCI + ADSCs groups, DiI-labeled cell count showed that 0.006% of transplanted ADSCs and MT-ADSCs were present in the injured area. In addition, independent t-test analysis demonstrated that the number of DiI-positive cells at the site of SCI in the SCI + MT-ADSCs group (107.33 ± 5.61) was significantly higher than in the SCI + ADSCs group (63.83 ± 7.13) [t (10) = 4.792, P < 0.001] (Fig. 4).

### Differentiation of ADSCs and MT-ADSCs into neurons

**Fig. 5:**
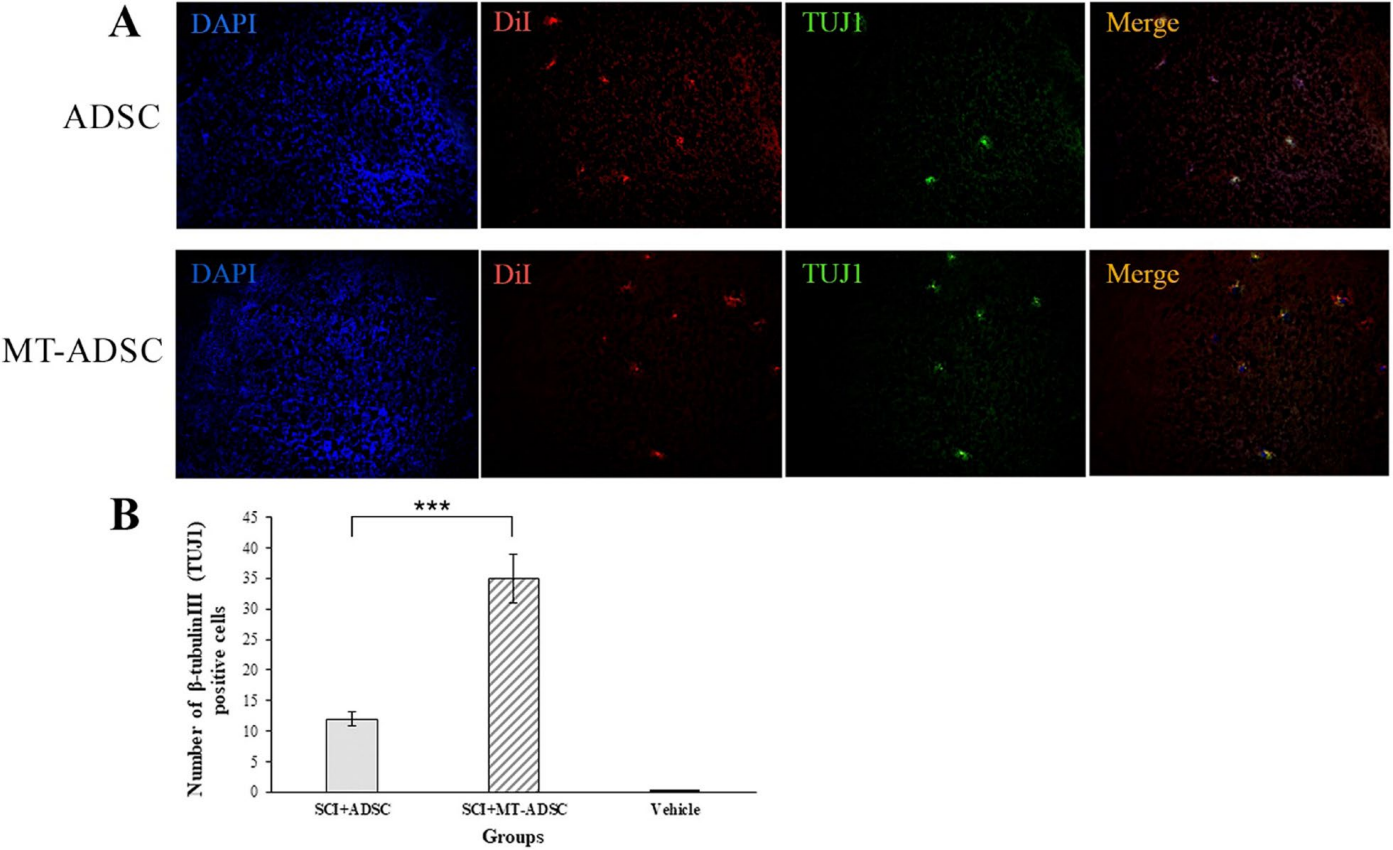
Differentiation of ADSCs and MT-ADSCs into neurons based on β-tubulinIII (TUJ-1) staining. **A** At the site of injury: the nuclei of ADSCs and MT-ADSCs labeled with DAPI; ADSCs and MT-ADSCs labeled with DiI; Neuron differentiation of ADSCs and MT-ADSCs labeled with β-tubulinIII. **B** Comparison of the number of β-tubulin III positive cells between Vehicle (SCI + DMEM), SCI + ADSCs, and SCI + MT-ADSCs groups demonstrated that MT-ADSCs differentiated into neurons more than ADSCs at the site of SCI (***P < 0.001); (Magnification: 20×)

In this study, β-tubulinIII (TUJ-1) staining was performed in the Vehicle, SCI + MT-ADSCs, and SCI + ADSCs groups to evaluate the differentiation of ADSCs and MT-ADSCs into neurons. According to the results, β-tubulinIII (TUJ-1) was expressed in both experimental groups transplanted with ADSCs and MT-ADSCs. Moreover, the number of β-tubulinΙΙΙ stained cells at the SCI site in the SCI + MT-ADSCs group (35.00 ± 4.00) was statistically significantly higher than in the SCI + ADSCs group (9.81 ± 1.15) [t (10) = 5.824, P < 0.001] (Fig. 5).

### Differentiation of ADSCs and MT-ADSCs into oligodendrocyte lineage cells

**Fig. 6:**
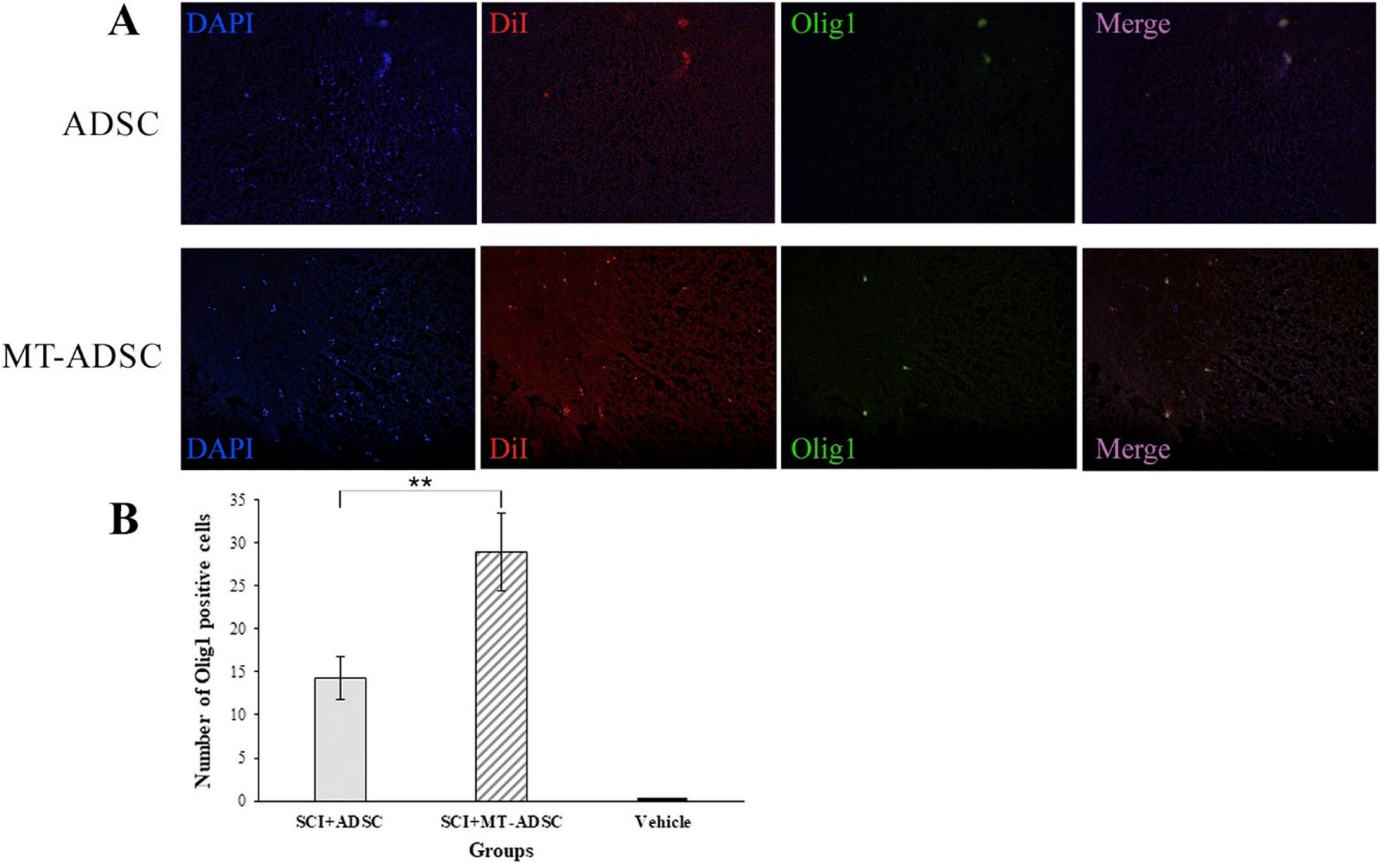
Differentiation of ADSCs and MT-ADSCs into oligodendrocyte lineage cells based on Olig1 staining. **A** At the site of injury: the nuclei of ADSCs and MT-ADSCs labeled with DAPI; ADSCs and MT-ADSCs labeled with DiI; Differentiation of ADSCs and MT-ADSCs into oligodendrocyte lineage cells labeled with Olig1. **B** Comparison of the number of Olig1 positive cells between Vehicle (SCI + DMEM), SCI + ADSCs, and SCI + MT-ADSCs groups showed that MT-ADSCs differentiated into oligodendrocyte lineage cells more than ADSCs at the SCI site (**P < 0.01); (Magnification: 20×)

Olig1 staining was accomplished in the Vehicle, SCI + MT-ADSCs, and SCI + ADSCs groups to investigate the differentiation of ADSCs and MT-ADSCs into oligodendrocyte lineage cells. The results suggested that Olig1 was expressed in both experimental groups transplanted with ADSCs and MT-ADSCs. Also, the number of Olig1-labeled cells at the SCI site in the SCI + MT-ADSCs group (29 ± 4.52) was significantly higher than in the SCI + ADSCs group (14.33 ± 2.48) [t (9) = 2.978, P < 0.01](Fig. 6).

### Differentiation of ADSCs and MT-ADSCs into astrocytes

**Fig. 7:**
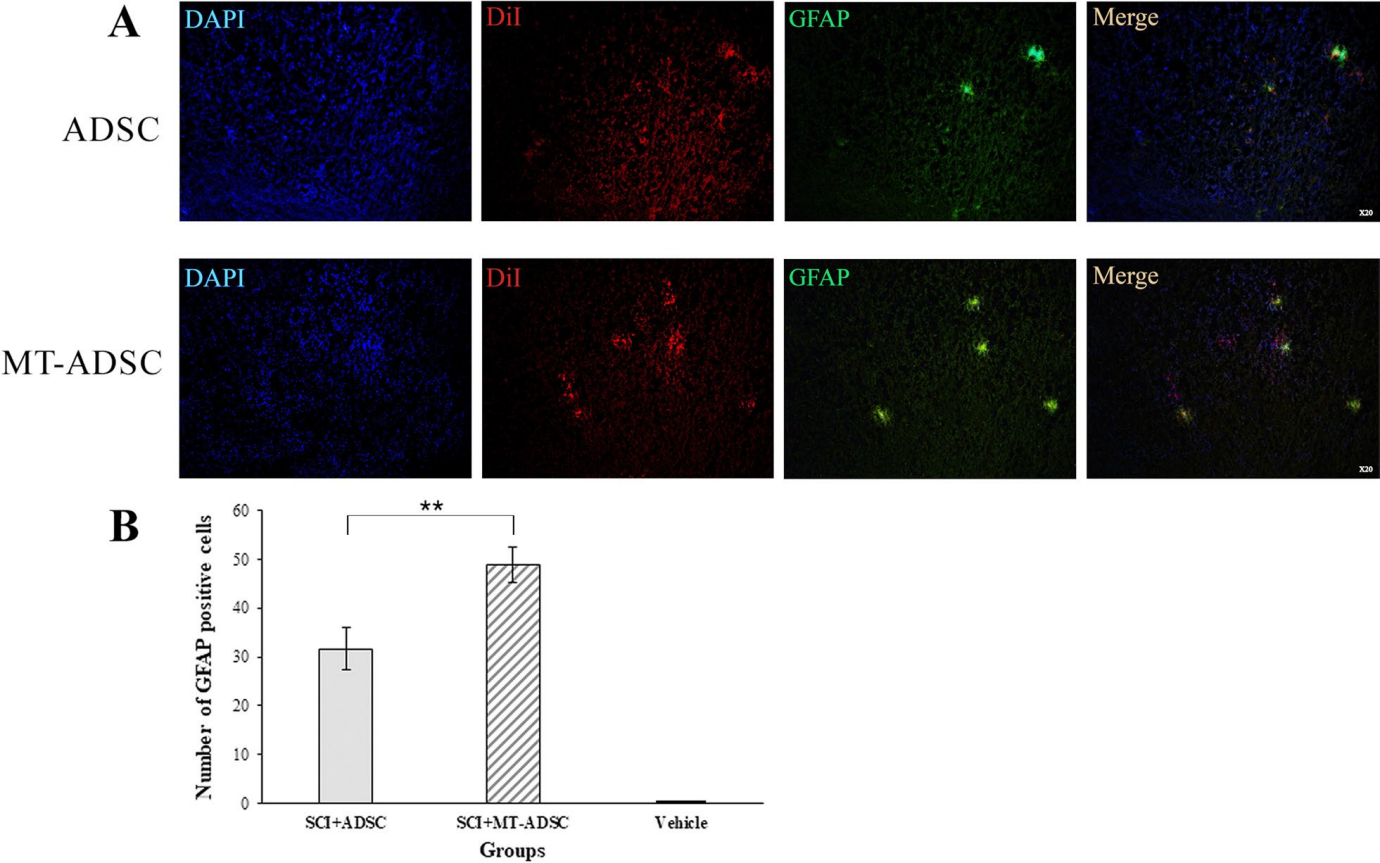
Differentiation of ADSCs and MT-ADSCs into astrocytes based on GFAP staining. **A** At the site of injury: the nuclei of ADSCs and MT-ADSCs labeled with DAPI; ADSCs and MT-ADSCs labeled with DiI; Astrocytes differentiation of ADSCs and MT-ADSCs labeled with GFAP. **B** Comparison of the number of GFAP positive cells between Vehicle (SCI + DMEM), SCI + ADSCs and SCI + MT-ADSCs groups revealed that MT-ADSCs differentiated into astrocytes more than ADSCs at the site of SCI (**P < 0.01); (Magnification: 20×)

GFAP staining was carried out in the Vehicle, SCI + MT-ADSCs, and SCI + ADSCs groups to evaluate the differentiation of ADSCs and MT-ADSCs into astrocytes. Based on the results, GFAP was expressed in both experimental groups transplanted with ADSCs and MT-ADSCs. However, the number of GFAP stained cells at the SCI site in the SCI + MT-ADSCs group (48.83 ± 3.71) was significantly higher than in the SCI + ADSCs group (31.66 ± 4.36) [t (10) = 2.994, P < 0.01](Fig. 7).

### BBB score evaluation

**Fig. 8:**
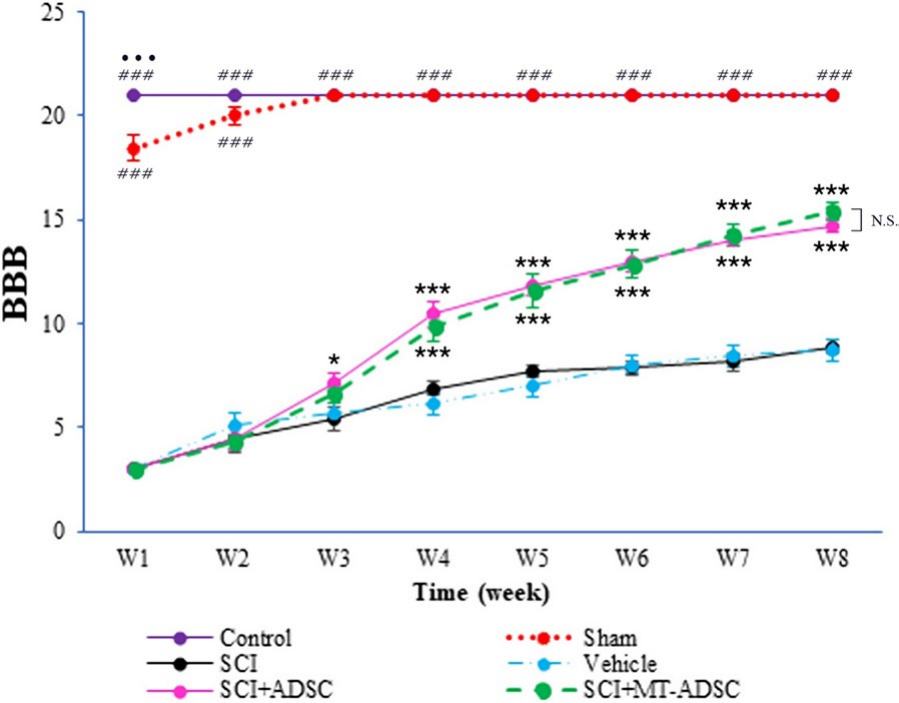
Evaluation of the BBB scores among Control, Sham, Model(SCI), Vehicle(SCI + DMEM), SCI + ADSCs, and SCI + MT-ADSCs groups. After the cell transplant, during the week1 significant differences were observed between the Control and other groups(P < 0.001); Within the week4, SCI + MT-ADSC and SCI + ADSC groups had higher scores than SCI and vehicle groups(P < 0.001); During weeks 5–8, significant differences were found between the SCI + MT-ADSC and SCI + ADSC groups and the SCI group(P < 0.001). The SCI + ADSC and SCI + MT-ADSC groups had no significant differences during the experimental period. ••• indicates P < 0.001 vs. the Sham group, ### indicates P < 0.001 vs. the SCI, Vehicle, SCI + ADSC, and SCI + MT-ADSC groups, * indicates P < 0.05 vs. the SCI group, *** indicates P < 0.001 vs. the SCI group

Based on the results, in the first week following the cell transplantation, there were significant differences between the Control and other groups (Week 1: f (5,41) = 1205.28, P < 0.001). Interestingly, within the fourth week, the BBB scores of the SCI + MT-ADSCs and the SCI + ADSCs groups were significantly higher than those of the SCI and vehicle groups (Week4: f (5,41) = 219.79, P < 0.001). Furthermore, a significant difference was noted between the SCI + MT-ADSCs and SCI + ADSCs groups and the SCI group in the fifth (Week 5: f (5,41) = 175.06, P < 0.001), sixth (Week 6: f (5,41) = 210.263, P < 0.001), seventh (Week 7: f (5,41) = 226.895, P < 0.001), and eighth (Week 8: f (5,41) = 256.03, P < 0.001) weeks following the cell transplantation. However, no significant difference was observed during the experimental period between the SCI + ADSCs and SCI + MT-ADSCs groups (Fig. 8).

## Discussion

Our study suggested that adipose-derived stem cells (ADSCs) pretreatment with melatonin improves the viability and proliferation of ADSCs. Accordingly, ADSCs reached a 40–50% density in 4–5 days, while the compressibility rate in MT-ADSCs was 80–90% before injection. The effect of melatonin on increasing the proliferation and viability of ADSCs has been well documented in the literature. Liao et al.'s study on the pre-conditioning of ADSCs in liver cell transplantation showed that melatonin could increase cell viability and prevent apoptosis in oxidative injury [2]. Similar findings have been reported for the effect of melatonin on ADSCs transplantation in a rat model of myocardial infarction [22, 23]. Several mechanisms have been proposed for these effects of melatonin, one of which is the removal of reactive oxygen species (ROS) and the application of antioxidant roles. Growing evidence has shown that oxidative stress leads to premature death, slow deviation, and genomic instability of cells, which ultimately diminishes transplant efficacy [23]. ROS can also impair the adhesion ability of mesenchymal stem cells and increase apoptosis by disrupting cells and matrix interactions called anoikis [22]. Liao et al. suggested that melatonin could decrease the production of these species and prevent ADSCs' death by inhibiting the activity of NADPH oxidase (NOX), which is the main source of ROS in cells [24]. Moreover, Ma et al. reported that melatonin could accelerate the clearance of reactive oxygen species by inhibiting the P53-CypD signaling pathway. The P53-CypD signaling pathway is a major pathway activating stress responses and apoptosis [23].

The role of inhibiting apoptosis has been demonstrated in other studies. For example, it has been shown that melatonin can significantly increase the expression of apoptotic inhibitor genes BCl-2 and Cyclin-D1. It also undermined the expression of the BAX pro-apoptotic gene, thus preventing cell death and increasing cell proliferation and survival under mounting oxidative stress [2]. The study of ADSCs transplantation in human kidney cells by Zhao et al. revealed that melatonin increased the expression of anti-apoptotic and pro-survival genes P-Erk1/2, SOD1, HO-1, and Bcl-2 [25]. In our study, the number of ADSCs pretreated with melatonin was more pronounced than that of non-treated ADSCs at the SCI site, which could be due to increased cell migration, enhanced cell proliferation, or decreased apoptosis. The role of melatonin in cell migration has also been demonstrated in other studies. For instance, Liao et al. reported that melatonin, as an antioxidant, has protective effects on cell migration and homing against oxidative injury [2]. This effect could be because of the recovering effect of melatonin on ADSCs’ adhesion capacity as well as heightened expression of CXCR4, a key receptor chemokine mediating cell migration and homing [2, 24].

Another challenge in ADSC transplantation is the differentiation of these cells into neurons. By definition, mesenchymal cells, if cultured properly, are expected to differentiate into osteogenic, adipogenic, and chondrogenic classes. However, researches show that they could be differentiated into neural categories by creating cultures under certain conditions [26]. Various protocols have been proposed to improve the differentiation of these cells into neurons [27]. In this regard, Santos Roballo et al. showed that the simultaneous culture of neurons and ADSC can differentiate these cells into neurons under in vivo conditions and reduce peripheral nerve regeneration following nerve damage [28]. This study argues that pre-conditioning ADSCs with melatonin can increase their differentiation into neurons, astrocytes, and oligodendrocyte lineage cells. To the best of our knowledge, this is the first study to investigate the role of melatonin in enhancing the differentiation of ADSCs into neurons. Ponchai et al., in their study on amniotic fluid-derived mesenchymal stem cells, suggested that melatonin can reinforce the differentiation of mesenchymal cells into neurons, especially dopamine-producing cells by interacting with two pathways, ERK and CaMKK2. It has been shown that the former pathway is mediated by the melatonin receptor, while the latter is independent of the melatonin receptor [29]. Contrary to our findings, in that study, melatonin inhibited the production of astrocyte cells [29], which could be due to different stem cell types and higher melatonin concentrations in our study. Further studies are warranted to shed light on the effect of different concentrations of melatonin as well as stem cell types on neuronal differentiation. The ability of ADSCs to differentiate into motor neurons, along with their anti-inflammatory and anti-apoptotic capabilities, make these cells an ideal candidate for mitigating disabilities following SCI [30]. Given that in SCI, loss of mobility is a main cause of disability, replacement of lost motor neuron cells and prevention of secondary damage to the remaining neuron cells can play a crucial role in improving a person's motor function [27]. A systematic review by Rafiei et al. also demonstrated that ADSC is able to improve the performance of motor tests, especially the Basso, Beattie, Bresnahan (BBB), irrespective of the type, severity, and place of injury, type of injection, and the number of injected cells [30]. In the present study, the results of the BBB test exhibited that eight weeks after transplantation, the motor function of the models improved significantly. Although studies have demonstrated that melatonin can improve the motor function of ADSC models [31], our results suggested that melatonin can effectively improve motor models compared to non-conditioned ADSCs but is unable to make a significant difference.

## Conclusions

In summary, we demonstrated that the pre-conditioning of ADSCs with melatonin enhances the number of engrafted cells and neuronal differentiation in the SCI site compared to the non-treated ADSCs. Our results also showed that although melatonin can effectively improve neurological functions in SCI compared to non-conditioned ADSCs, it cannot make a significant difference. Therefore, regarding the positive effects of melatonin at the cellular level, we suggest a future study design with a longer follow-up duration and higher doses of melatonin for elucidating the functional outcome of the agent.

## Methods

### Animals

42 eight-nine-week-old male Sprague–Dawley rats, each weighing 250–300 g, were obtained from the Animal Care Center of Guilan University of Medical Sciences. The rats were housed in an animal room with a constant temperature (23 ± 1 °C) and humidity level (50–60%) with a 12-h light/dark cycle and provided ad libitum access to a standard diet and water. The experimental protocols were approved by the Animal Experimentation Ethics Committee of the Guilan University of Medical Sciences (IR.GUMS.REC.1399.227) and were conducted in accordance with animal ethics guidelines of the national health and research institutes. The Rats were randomly divided into six groups (n = 7) as follows:

- Control group: This group of rats did not receive any treatment or injury.
- Sham group: These rats underwent surgical stress(laminectomy).
- Model group: These rats suffered only lesions without receiving any therapeutic agent(SCI).
- Vehicle group: DMEM was injected intravenously into this group of rats one week after SCI (SCI + DMEM).
- Lesion treatment A: ADSCs were injected intravenously into this group of rats one week after SCI (SCI + ADSCs).
- Lesion treatment B: melatonin pretreated ADSCs were injected intravenously into this group of rats 1 week after SCI (SCI + MT-ADSCs).

### Adipose-derived stem cell extraction

The white adipose tissue of the mouse epididymis was removed in antiseptic conditions, rinsed with saline, and then digested in filtered (0.20 μm syringe filter) 0.075% collagenase at 37 °C for 60 min. The enzymatic activity was inhibited by adding low-glucose DMEM containing 10% FBS and 1% penicillin–streptomycin, and the cells were pelleted by centrifugation at 1000 rpm for 7 min. Pellets were resuspended in a 160 mM ammonium chloride solution and incubated at room temperature for 3 min, then filtered (70-μm nylon mesh). Once again, cells were pelleted and resuspended in DMEM and cultivated in 75 cm2 flasks under 5% CO_2_. Medium changes were made every four days, and when the cultures reached 90% confluence, cells were sub-cultivated using 0.25% trypsin/EDTA(Sigma, USA).

### Flow cytometry analysis of adult mouse adipose-derived stem cells

The expression levels of positive (CD90, CD73, CD105, and CD44) and negative (CD45 and CD31) cell surface markers were assessed to confirm the isolated cells as ADSCs. Briefly, adult mouse ADSCs at the third passage times were harvested using 0.25% trypsin and 0.02% EDTA, then washed with 0.01 M PBS. Subsequently, 50-ml suspensions at a cell concentration of 10^6^/ml were utilized as the test set. The test sets were incubated for 20 min with anti-mouse CD105, CD90, CD73, CD45, CD44, and CD31 antibodies(BD bioscience, USA). Following twice-rinsing in PBS, the cells were run on an NxT Flow Cytometer (Attune™; Thermo Fisher Scientific, USA).

### Melatonin pretreatment

For melatonin pretreatment, after the third passage, the ADSCs were incubated with 5 μM melatonin (M5250-1G, Sigma, USA) for 24 h before injection [32, 33]. All pre-conditioned cells were rinsed three times with PBS to remove any residual melatonin before further analysis.

### Cell labeling

The ADSCs were resuspended with 1 μM CellTracker™ Cm-dil (Qcbio Science&-Technologies Co., Ltd., Shanghai, China) at a density of 1 × 10^6^/mL in a complete medium at 37 °C for 5 min, followed by incubation at 4 °C for 10 min, and rinsed with PBS for three times, and subsequently collected for cell transplantation.

### The spinal cord injury model

In this study, we adopted Lee et al.’s clip compression method to develop spinal cord injuries[34].

After anesthesia (ketamine 60 mg/kg and xylazine20 mg/kg), a Clip compression injury was performed on the 9-10th thoracic segments by removing their dorsal processes under sterile conditions. Sutures were then applied to close the wound. Following surgeries, the rats received postoperative care, including subcutaneous administration of Cefazoline (50 mg/kg) daily for 5 days. Also, urinary bladders were emptied twice a day by abdominal compression. Finally, animals were examined 3 days after spinal cord injury and were excluded if the BBB score was higher than three. In addition, five cross-sections of micro-straws were also made from the lesion to verify the model [34].

### Basso, Beattie, and Bresnahan (BBB) score

**Fig. 9:**
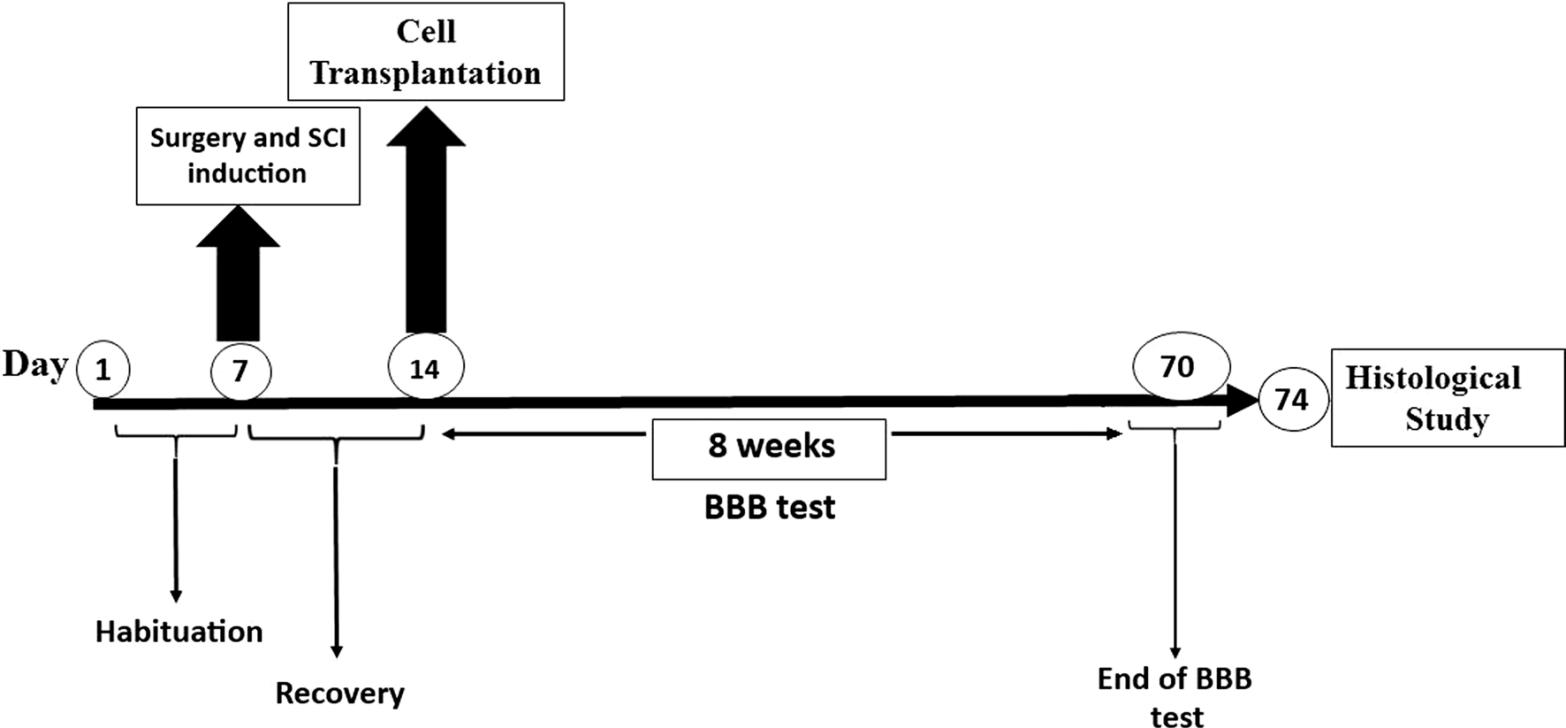
Diagram of the study

After cell transplantation, the BBB score was used weekly to monitor and evaluate animal neurological function [35] for up to 8 weeks (Fig. 9). BBB is the primary scale used to assess motor recovery in rats with spinal cord injury in studies conducted by MASCIS (Multicenter Animal Spinal Cord Injury Study) [36]. Based on the BBB score criteria, 21 scores were assigned (0 for no observable hind limb movement and 21 for normal locomotion). Rats with a score from 0 to 7 (early stage of recovery) showed little or no hindlimb movement; rats with a score of 8 to 13 (intermediate stage of recovery) exhibited uncoordinated steps, and rats with a score of 14–21 (late stage of recovery) displayed coordinated forelimb and hindlimb movement [37].

### Histopathological and immunohistochemistry studies

Sixty days after cell transplantation, the spinal tissues were collected to evaluate engrafted cell numbers and stained with anti-Olig1, anti-β-tubulin III (TUJ-1), and anti-GFAP antibodies to identify cells differentiation into oligodendrocyte lineage cells, neurons, and astrocytes, respectively. Briefly, after deep anesthesia, 4% paraformaldehyde in PBS buffer was used for perfusion. The spine tissues were then harvested and fixed using 4% paraformaldehyde. Following tissue processing, tissues were paraffin-embedded (Merck, Germany). Using a microtome, 5 µm-thick sections of spinal tissues were prepared. To calculate the number of implanted cells, three sections at a distance of every 20 sections from the epicenter of the lesion were selected per rat. When the sections were cleared and dehydrated, antigen retrieval was utilized. Following incubation with the blocking solution, spine sections were incubated with anti-Olig1, anti-β-tubulin III, and anti-GFAP mouse primary antibodies at 4 °C overnight. Next, the sections were washed with PBS and incubated using goat anti-mouse secondary antibody for 2 h at room temperature. After PBS washing, DAPI was used for counterstaining the nuclei for 30 min; then, the sections were mounted. Finally, labeled stem cells were observed directly by fluorescent microscopy and OLYSIA Bio Report Soft Imaging System. Cell count was carried out by calculating dying cells, the nuclei of which were stained with DAPI. Hematoxylin and Eosin(H&E) staining were also developed for the SCI confirmation.

### Statistical analysis

The results were expressed as mean ± standard deviation(SD). The Kolmogorov–Smirnov test was used to check the normality of values. Statistical analysis of BBB scores was performed by one-way analysis of variance(ANOVA), followed by Tukey post hoc for comparisons. Other data were analyzed by independent t-test. Statistical analysis was done using the IBM SPSS Statistics for Windows, version 26 (IBM Corp, Armonk, NY, USA), and differences were considered statistically significant when P < 0.05.

## Data Availability

The datasets generated and/or analysed during the current study are available from the corresponding author on reasonable request.

## Abbreviations

SCI: Spinal cord injury
ADSC: Adipose-derived stem cell
MT: Melatonin
IHC: Immunohistochemistry
ESC: Embryonic stem cell
BMSC: Bone marrow stem cell
GAP-43: Growth-associated protein 43
BDNF: Brain-derived neurotrophic factors
NGF: Nerve growth factors
ROS: Reactive oxygen species
ERS: Endoplasmic reticulum stress
BBB: Basso, Beattie, and Bresnahan
MASCIS: Multicenter Animal Spinal Cord Injury Study
Olig1: Oligodendrocyte lineage gene 1
GFAP: Glial fibrillary acidic protein
NOX: NADPH oxidase
SD: Standard deviation
ANOVA: Analysis of variance
MSC: Mesenchymal stem cell
